# The Strategies of Picture Books as a Mode of Health Communication for Young Children with Coeliac Disease

**DOI:** 10.3390/children12050530

**Published:** 2025-04-22

**Authors:** Lydia McKeon, Jessica Gildersleeve, Amy B. Mullens

**Affiliations:** 1 School of Psychology and Wellbeing, Centre for Health Research, University of Southern Queensland, Toowoomba, QLD 4350, Australia; w0027634@umail.usq.edu.au (L.M.); amy.mullens@unisq.edu.au (A.B.M.); 2 School of Humanities and Communication, Centre for Health Research, University of Southern Queensland, Toowoomba, QLD 4350, Australia

**Keywords:** coeliac disease, coping, resilience, health communication, health narratives, young children

## Abstract

**Background/Objectives:** Coeliac disease, a chronic and lifelong health condition, is one of the most common autoimmune diseases. However, it is also one of the most under-recognised conditions, and emotionally and cognitively appropriate materials are especially lacking for young children and their families who are coping with this disease. Effective health communication is essential for educating and supporting children living with coeliac disease as well as their families and communities. Picture books can serve as useful and accessible educational and health promotion tools, promoting adaptive coping strategies for dealing with a potentially traumatic condition. **Methods:** This study aimed to fill a critical gap in the literature by examining a range of picture books (*n* = 9) aimed at children three to eight years of age diagnosed with coeliac disease. Reflective thematic and structural narrative analyses were applied to explore strategies and themes used in these books and how they align with the current literature on developing coping through children’s narratives. **Results:** Four themes were developed and measured against an existing model of coping narratives to find a more specific model that recognises the specific concerns of coeliac disease. The four themes found were Information Provision; Promotion of CD Management; Anxiety and Hypervigilance Reduction, with two subthemes of Validating Feelings and Reducing Concerns; and Community and Connection. **Conclusions:** The findings have likely implications for the following applications: incorporation into clinician training (as a therapeutic and health promotion intervention), support within schools, authors of similar books for children coping with chronic illness, and coping approaches for individuals/families to promote health literacy/support regarding living with coeliac disease.

## 1. Introduction

Children’s story and picture books can provide an ideal opportunity for delivering health education regarding effective ways of coping with the diagnosis of a chronic health condition and its ongoing management or treatment. One of the most difficult experiences for a young child (i.e., ages three to eight years) is learning to cope and adapt to being diagnosed with a lifelong “silent” condition such as coeliac disease. Picture books can be a useful resource in supporting a child’s learning, as well as that of their families, thereby creating an important link between the narrative, the reader, and the child [1]. Picture books largely cover this age range as this is a period of rapid change and development, particularly in language and learning [2,3]. Picture books can also be a vital coping mechanism by providing an outlet and explanation for emotions [4].

Coeliac disease (CD) is a chronic digestive and autoimmune illness that stops the body from processing gluten, a protein found in wheat, rye, barley, and oats [5]. Gluten is included in many common foods, such as pasta, bread, and pastries, as well as other food items such as sauces, dressings, and confectionaries. When a person who is living with CD consumes gluten, the lining of the small intestine is damaged, causing chronic inflammation of the villi (tiny projections on the lining of the small intestine), which reduces the absorption of nutrients and minerals, leading to nutritional deficiencies. Even small amounts of gluten can cause harm and severe symptoms. Approximately 1 in 70 Australian children have CD, and approximately 1 in 100 people have the disease worldwide, making it one of the most common autoimmune diseases. However, it is also one of the most under-recognised medical conditions, with around 80% of cases undiagnosed [6]. Children with a family history of CD are at higher risk of developing the disease themselves, approximately ten times higher than someone without a family history [7].

A range of symptoms accompany CD, which can vary from person to person [8]. Some of the more common symptoms include abdominal pain, diarrhoea, bloating, vomiting, weight loss [9], persistent low energy and headaches, stomach rumbling and growling, and hunger pain [10]. Younger children can present with different symptoms compared to older children and adults, with symptoms such as irritability, suboptimal growth, failure to thrive, abdominal distention and pain, malabsorption, delayed puberty, decreased bone density, dental enamel defects, dermatitis herpetiformis, iron deficiency anaemia, hepatitis, and anorexia [11]. Some individuals may notice severe symptoms immediately after eating foods containing gluten, while others may experience symptoms 24 to 48 h later [12]. Some children can have no noticeable symptoms at all; however, damage to the small intestine is still apparent [11]. A study by Lal et al. found over 11% of children diagnosed with CD were asymptomatic [13].

Several psychological implications are associated with CD, including an increased risk of mood, anxiety, eating, behavioural, and intellectual disorders; attention-deficit/hyperactivity disorder (ADHD); substance misuse; autism-spectrum disorder (ASD); and psychotic disorders [9,14]. Even before receiving a diagnosis of CD, symptoms caused from eating gluten can contribute to the increased risk of psychological issues. This is related to the constant feeling of being unwell, a long period of uncertainty and frustration, and the looming threat of dealing with a lifelong illness and treatment after receiving a diagnosis [9]. Strong associations have been found between children living with CD and anxiety problems, oppositional defiant problems, and aggressive behaviour [15].

If left untreated, CD can also lead to long-term health problems, such as liver disease, osteoporosis, infertility, and a higher risk of developing other autoimmune conditions [12]. There is a smaller increased risk of specific types of cancer such as lymphoma of the small intestine. The only current evidence-based treatment is to follow a lifelong gluten-free diet (GFD), which allows the small intestine to recover [6]. However, due to the restrictions of a GFD, there is also the potential for adverse nutritional outcomes. Being on a GFD long-term can lead to higher rates of overweight/obesity and metabolic complications such as cardiovascular disease and fatty liver disease [16], adding to the already extensive list of challenges in maintaining adherence to CD treatment.

Picture books and stories for young children are an important source of language, conceptual, and educational development [17]. Stories can foster connection with others, socialisation, and aid in forming the reader’s identity [18]. Storytelling is also a powerful way to cope with trauma, stress, and psychological difficulties and to help to make meaning of the world [19]. Engaging with stories can also help to normalise mental health conditions and chronic diseases [20]. Stories can thus be a powerful tool to increase health information and health literacy and may also serve functions such as normalisation and increased self-efficacy [17].

### 1.1. Health Communication and Coeliac Disease

The current literature has found that continuous self-management can lead to improved health outcomes in people living with chronic diseases. Self-management includes acquiring the ability to perform a range of skills such as being flexible; carrying out plans; decision making; and problem solving to support learning. Self-management is a crucial approach for managing chronic conditions such as CD, in which adherence to a lifelong GFD is the sole treatment [21]. Teaching self-management is possible from an early age by offering appropriate strategies for disease prevention and management throughout life and building the child’s capacity to cope with daily challenges. Building these coping strategies has shown positive impacts on subsequent health and health behaviours [22].

To identify previous research focused on self-management skills for children under the age of eight diagnosed with a chronic condition, interventions for young children with type 1 diabetes (T1D), another autoimmune condition, were explored. A diagnosis of T1D has similar implications for children and families as a diagnosis of CD, with the requirement of adapting to a new lifestyle, maintaining a strict diet, and the daily management challenges a diagnosis brings [23] for a lifelong condition. This literature showed that the parents of young children are mostly responsible for their child’s daily management of T1D, which includes diet regulation to prevent the onset of acute and chronic complications. According to Streisand and Monaghan, some interventions included social support, coping skills training, and stress management for the parents [23]. This resulted in several improvements, including social support, quality of life (QOL), wellbeing, and overall improvements in children’s diabetes-related health levels [24]. Catarino et al. found that self-management programmes for chronic disease in children and adolescents were used to educate children for problems in their daily life [25]. These programmes include activities designed to achieve knowledge about T1D and the acquisition of skills. This emphasises the role of patient education in health care activities and disease self-monitoring [25] and shows that an understanding of CD is likely to have a positive impact on treatment adherence [26] and subsequent health outcomes.

#### 1.1.1. Adherence to a Gluten-Free Diet

Adhering to a GFD requires executive functioning skills such as planning, prior organising, impulse control, and flexibility to adapt to frequently changing situations [27]. While for children living with CD, this responsibility is mostly assumed by parents, the ability to self-manage will be required as this responsibility shifts from the parents to the child as they get older, increasingly so towards adolescence. Having this ability and learning how to cope with the difficulties associated with CD is important for chronic disease management [21]. Adherence to a GFD has been found to increase when the individuals involved have a thorough understanding of CD [26,28], and younger children have shown to be more likely to comply with the diet than older children [29]. Parents’ knowledge of CD has also been found to positively contribute to the child’s adherence to a GFD. Further, when the responsibility is shared between the child and parents, GFD adherence is higher [30].

Adherence to the GFD can also be impacted by the self-efficacy and outcome expectancies children acquire for maintaining the diet [31]. Bandura’s social learning theory suggests that children’s behaviours are reinforced through observational learning and imitation of others [32]. The observation of others performing a task, such as the preparation or consumption of gluten-free food, can influence judgements about their own capabilities and affect children’s expectancies for this behaviour [32]. Young children have a lower impulse control compared with older children and adults [33] and find it difficult to prepare for situations in which assured safe food may be challenging to find. They also may fail to recognise, consider, or understand the potential negative health effects of consuming gluten [31], especially when the consequence (sickness) that occurs after eating gluten is delayed. This has a detrimental effect on adherence to the GFD as the severity of its impacts is not obvious.

#### 1.1.2. Psychological Implications and Coping Behaviours

Strict adherence to a GFD can be challenging due to several reasons, including social isolation, risk of cross-contamination, and the high cost and taste of gluten-free foods, as well as being perceived as requiring continual sacrifices. The GFD has shown to have a significant negative impact on QOL in social settings as well as psychological distress and anxiety related to diagnosis [28,29]. Individuals living with CD are more likely to experience depression, anxiety, lower health-related QOL, and poorer psychological wellbeing than those without CD. The impact on social interactions can result in feelings of isolation and exclusion, social phobias and depression [34,35], and the thought of being a “burden to others”. Coping with CD involves coping with constant food restrictions and challenging social situations, including being “different” to peers and not being able to go to a social gathering without the fear, stress, and anxiety of being able to eat safely or of becoming ill [9,28].

Although worry and fear at milder levels can be adaptive towards health-protective behaviours [36], the potential consequences from the constant and very real fear of possible gluten exposure can lead to heightened anxiety and hypervigilance [35,37] for a person with CD, which leads to regular check-ins with their body, frequently assessing how their digestive system is reacting to food. This develops a high attentional bias toward bodily functions and sensations and can result in increased anxiety, depression, and other mental distress. Hypervigilance regarding food and eating also has social ramifications of stigma and isolation and can lead to disordered eating behaviours including avoidant or restrictive food intake [37]. Even if adherence to a GFD is higher, psychological outcomes and QOL have still been found to be lower in children living with CD [35]. Research has shown that implementing coping strategies can improve the psychosocial wellbeing of patients diagnosed with CD [38]. If an individual has the ability or skills required to control or manage a situation, they are more likely to successfully apply positive approach strategies [39]. Young children can apply effective coping strategies and understand emotion-regulating strategies if provided with age-appropriate methods [40].

### 1.2. Stories as a Means of Coping

Storytelling has been well established as an effective tool in providing children with new information about the world, enhancing language abilities, and further developing vocabulary, both in the home and in school environments [41]. Matching the ideas within stories to children’s real problems allows the child to feel comfortable and safe in confronting their own difficult situations, providing emotional security and healthier ways of dealing with internal struggles, life adversities, and stressors [42]. The importance of including diverse characters so that children can identify with the protagonist has also been highlighted [19,43].

Many studies have demonstrated a positive relationship between stories/storytelling and enhanced coping abilities, and stories have been assessed as a means of coping in a range of individuals and situations. Some of these include children coping with hospitalisation [44], immunisations [45], and the COVID-19 pandemic [46]. Children’s stories containing coping strategies were used to answer children’s questions, clarify their doubts, and help to normalise their emotions and understand the grieving process [43]. The act of storytelling has also shown its positive effects on children’s resilience, which is a fundamental aspect of mental health and coping that provides individuals with the skills to effectively recover from challenges [47] and build resilience.

### 1.3. Children’s Stories as a Health Strategy

Storytelling can be effectively applied to promote health-related behaviours in a range of ways and is a comfortable and familiar way of exchanging information. This type of message engages individuals’ brains at a conscious and rational processing level. The more individuals identify with the characters/contexts in stories, the more they perceive the health message to be accurate, useful, and believable [19]. Children’s stories have been found to play a fundamental role in helping children learn about health and coping strategies in a developmentally appropriate and accessible format [48]. Picture books with messages about dietary behaviours and feeding strategies were found to commonly portray specific behaviours as well as strategies to deal with problem behaviours [49]. Storytelling activities in school settings provide an innovative didactic experience capable of building children’s health knowledge and promote students’ global wellbeing. Children’s stories can also be used to empower and motivate children towards the adoption of health behaviours; however, story content must relate to the child’s situation and feelings as much as possible [50]. Children’s stories can be a positive way of providing health strategies to help young children coping with health-related issues and a successful format for portraying related knowledge to increase understanding for the children. There is, however, a complete scarcity of research focused on children’s stories as a health strategy for young children diagnosed with CD, which represents a critical gap to be addressed.

### 1.4. Model of Coping Narratives

The model of coping narratives (MCN) was developed by Gildersleeve et al. after examining a range of multimedia resources focused on the COVID-19 pandemic and targeted towards Australian children under the age of five years [46]. The aim of this study was to develop a greater understanding of appropriate narrative models for the target audience. Using thematic analysis, Gildersleeve et al. identified four core themes from within the range of pandemic narratives [46]. These four themes were Information Provision, which included the identification of health and hygiene instruction, explanatory knowledge about the virus, and preventative measures; Promotion of a New Normal, which included everyday routine changes, challenges or adjustments, and promotion of wellbeing and self-care; Anxiety Reduction, which included acknowledging children’s feelings and attempting to reduce fear and anxiety; and Community and Connection, which included promoting the importance of social support in building coping and resilience. A connected hierarchy of coping strategies was also discovered within the four themes in which each theme feeds into the next. Gildersleeve et al. identified that the most effective narratives are inclusive of all four themes, completing the cycle of instruction for children’s development of coping strategies [46].

### 1.5. The Current Study

Due to the developmental capacity in children under the age of eight years, parents and caregivers take primary responsibility for diet and disease management [23]. Therefore, parental knowledge of CD is vital for diet adherence and to provide better awareness for their children [28]. Previous research on adherence to a GFD for young people is limited but points out that young children require not only the help of their parents in maintaining strict adherence but many other parties within the child’s community. The acquisition of self-management skills and adherence in children is a process that requires the support of the family, health professionals, and the community [51]. Although research on CD is increasing, there are still many gaps to be explored. The focus on increasing awareness and knowledge for children and families, peers, and educators is required to provide young children and their families with the resources required to manage, as well as having the ability to use successful behaviours to increase overall QOL and wellbeing. Based on the research relating to children’s books as a successful format in delivering coping and health-related strategies for young children, with the help of the child’s support system, children’s picture books based on CD seem like a logical resource. The literature thus far does not yet examine picture books designed for children with a diagnosis of CD.

This study aims to help close this gap and add to the recent literature focused on how stories and age-appropriate narratives can be used to aid in developing coping strategies in children and young people experiencing trauma and health-related diagnoses. This study may lead to the development of age-appropriate health promotion used as a means of coping for individuals and families, provide support within the school system, as well as provide a tool for use by health professionals for therapeutic intervention and clinician training. This paper examines a selection of picture books aimed at children ages three to eight years diagnosed with CD. Using the MCN developed by Gildersleeve et al. [46], this paper analyses these books in terms of the provision of health education for children and how they align with the current literature. It seeks to identify the narrative strategies and themes used in picture books aimed at young children diagnosed with CD, consider how this aligns with or extends our previous understanding of chronic illness health communication, and suggest how this information can be used to promote health education for young children with CD.

## 2. Method

### 2.1. Data Collection

For this study, two methods under the narrative umbrella were used: thematic narrative analysis, which examines what is written; and structural narrative analysis (SNA), which focuses on how the story is told. To do so, this study relies primarily on Braun and Clarke’s reflexive thematic analysis (RTA) [52]. The data for this study were obtained by conducting a search to find available picture books aimed at young children between the ages of three and eight diagnosed with CD. The following search terms were used: “picture book”, “children stor*”, “child book”, “coeliac disease”, and “celiac disease”. The search began 1 March 2024 and ended on 22 May 2024, resulting in a collection of 40 books that initially met the criteria. Due to the updated and recent awareness of CD and its impacts, any books published earlier than the previous ten years were excluded, as well as any books that included information on food allergies as well as CD due to CD being an autoimmune disease rather than an allergy and the arising differences in how gluten attacks the body [53]. Following this, any books published outside of Australia and the United Kingdom were excluded. Following the exclusion criteria, the final selection of picture books selected for data analysis contained nine books (Table 1).

### 2.2. Data Analysis

Once the picture books were selected, RTA was used to identify common themes within each of the CD books, followed by SNA. The first step in this process included the researcher being completely immersed in and familiar with the dataset, one item at a time. This was followed by critically and reflexively engaging with the narrative within the books and taking initial notes throughout. The next step involved searching for segments within the data deductively, looking for relevant descriptions and making codes that aligned with the four themes discovered and included in Gildersleeve et al.’s MCN [46]. These themes included Information Provision, Promotion of a New Normal, Anxiety Reduction, and Community and Connection [46]. Following this, we inductively reviewed the data, searched for narratives relevant to the research questions, and wrote codes to subjectively create new meanings from the dataset specific to this study. Themes were then identified and generated from the codes and then refined further to define each theme more clearly [52]. The final step of SNA was applied to find similarities and differences between models within the stories and how they map onto previous models. This final step included considering the parts and themes developed separately and then as a whole to make meaning from the story and to identify how the stories were told [63].

### 2.3. Reflexivity

RTA lends itself to the incorporation of reflexivity and to managing any possible assumptions and biases within the analysis process. All authors collaboratively contributed professional and personal knowledge and expertise within the realm of CD/chronic health conditions, health literature, children’s literature, clinical/health psychology, and coping narratives, allowing the data to be critically approached from the position of insider and outsider research and to subjectively positively enhance the findings [64].

## 3. Results

Through RTA of the CD picture books (*n* = 9), Gildersleeve et al.’s MCN [46] was applied to measure how these books align with this model. It was found that the model provided a framework that enabled adequate analysis for how these books approached coping with CD. Table 2 outlines the deductive RTA, inclusive of the characteristics within each CD picture book for each theme within the model.

### 3.1. Information Provision

Most of the resources (*n* = 8) included in this study utilised Information Provision. This included identification of CD, explanations of what gluten is and where it can be found, what symptoms can occur from eating gluten with a CD diagnosis, how CD is diagnosed, and how to treat it. Four of the resources included all aspects of Information Provision in some way, whereas the remaining five resources only mentioned some or limited aspects of information, for instance, *How Hayden Found Her New Favourite Foods* included only that Hayden’s “tummy hurt” from eating gluten [60]; however, there was no further information explaining what gluten or CD is. Some of the resources included more thorough information: for example, *I Have Coeliac Disease: Autumn’s Story* included a comprehensive explanation within the story about where the CD genes come from and what is involved in CD testing and diagnosis. Other resources contain less extensive information: for example, *Little Jak the Silly Yak at Tara’s Birthday Bash* included minimal information on CD, stating only that “when he eats gluten, his tummy disagrees” and eating gluten “can make Jak feel quite poorly” [59], with no other specificity provided. This resource did, however, include a gluten-free recipe at the end of the book, which was also a feature of three other resources.

### 3.2. Promotion of a New Normal

Seven of the nine resources included information promoting the “new normal” regarding managing and adapting to living with a diagnosis of CD. This included information on how to adhere to a GFD, being careful about cross-contamination, and how and where to find gluten-free foods. Most of the resources in this category focused on there being plenty of gluten-free options available in order to promote change in children living with CD. For example, *How Hayden Found Her New Favourite Foods* encourages adapting to a new diet by explaining that there are plenty of recipes that taste good. Some of the resources were limited in the information included within this category, such as *Celia’s Coeliac Story*, which only makes mention of how to make gluten-free food exciting, suggesting the taste of gluten-free food is the only challenge to be addressed for someone living with CD, while *Gluten Tootin’* vaguely states there is lots of tasty food the character in the book can eat, without identifying these. Label-reading and knowing which foods can be consumed were discussed in five of the resources. For example, *Little Jak the Silly Yak Has Silly-Yak Disease* mentions that gluten is, “in so many things. Just check the label on the back, even the chicken wings. For wheat can be in all manner of food, you just can’t be too careful” [57].

Explaining the importance of cross-contamination was found in only three of the resources in this category; *Little Jak the Silly Yak: The Apple Pie Quest* states that “it can trigger many things, in as little as a crumb. When gluten-free food is prepared near anything that’s not, it will be contaminated. The whole, entire lot!” [58]. *I Have Coeliac Disease: Autumn’s Story* is more thorough in discussing cross-contamination by providing more detailed instruction. Two resources included information about being prepared and bringing special gluten-free food when going to a birthday party or an event. For example, *I Have Coeliac Disease: Autumn’s Story* discusses being prepared by bringing gluten-free food to parties and camps, while in *Little Jak the Silly Yak at Tara’s Birthday Bash*, the protagonist prepares a range of gluten-free treats before going to a birthday party so he will not miss out.

### 3.3. Anxiety Reduction

Most of the resources (*n* = 8) to some extent attempt to validate the child’s feelings and reduce anxiety by acknowledging that maintaining a GFD is difficult. However, even where it is provided, such acknowledgement is minimal and does not go further in exploring or recognising the character’s or reader’s feelings. For example, *Sophie Doesn’t Eat Gluten* states that “sometimes Sophie feels sad that she can’t eat the same thing as her friends”; however, “Sophie feels happy that she gets to eat yummy gluten free cakes and bread” [54]. Some of the resources (*n* = 3) attempt to validate the difficulties of the GFD; however, this is mostly focused on suggesting that the diet is difficult only because of the perception that the food does not taste nice. Only one resource discusses the difficulties of going to social events or outings and the possibility of there not being anything gluten free to eat. Three of the nine resources do not include any validation of feelings before attempting to reduce anxiety. Two resources provided anxiety reduction attempts by including practical coping strategies, such as planning ahead for social activities/events, discussed in *Little Jak the Silly Yak at Tara’s Birthday Bash*. *I Have Coeliac Disease: Autumn’s Story* suggests taking food when going to a party; however, there is no further explanation of why or how this could be achieved.

### 3.4. Community and Connection

Several of the resources (*n* = 6) recognised the importance of Community and Connection in supporting children who are living with CD. Two of the resources promote connection with friends through safety and support: for example, in *Sophie Doesn’t Eat Gluten*, Sophie’s friends help keep her safe by checking food with adults and not sharing food with her if they are unsure. In *How Hayden Found Her New Favourite Foods*, Hayden finds and cooks new recipes with the help of her mother after connecting with an online CD community. Three resources encourage inclusivity with friends and family, for example, in *Little Jak the Silly Yak at Tara’s Birthday Bash*, Jak feels accepted at a birthday party with his friends when all of the food provided is gluten free, meaning Jak could eat the same as everyone else. Two of the resources provide the possibility for children to connect with the character by initially identifying other defining characteristics before introducing the protagonist’s CD diagnosis. Three resources suggest the importance of making other people in the characters’ close community aware about what they can and cannot consume. *I Have Coeliac Disease: Autumn’s Story* states that “teachers need to be aware, if they have any coeliac children in their class then food and drink must be gluten free” [55]. However, there is the risk of confusion with this statement due to the difference between the reactions of someone living with CD or a gluten allergy and that the most appropriate course of action for a child with CD in a classroom is simply to ensure that no children share food.

### 3.5. Summary of Findings

The resources analysed in this study followed a similar pattern to the resources analysed in the study by Gildersleeve et al. [46]. However, when inductively analysing the present resources, it became evident that a more specific CD model was possible to further highlight some of the important aspects found within the CD resources and the current literature. The following four themes were found: Information Provision; Promotion of CD Management; Anxiety and Hypervigilance Reduction, with two subthemes of Validating Feelings and Reducing Concerns; and Community and Connection.

## 4. Discussion

### 4.1. Information Provision

According to Gildersleeve et al., stories that begin the narrative by providing information on the problem and completing the hierarchy of stages are the most effective narratives in developing effective coping strategies for children [46]. This theme, Information Provision, was found to be consistent with the Gildersleeve et al. model [46], as most of the picture books began with introducing some sort of CD or gluten-related information. Consistent with the literature, obtaining an understanding and knowledge of CD is beneficial in adhering to treatment and health guidelines [26]; therefore, being provided with the necessary information specific to this diagnosis is essential to being able to manage CD. *I Have Coeliac Disease: Autumn’s Story* is thorough in describing CD and gluten and the only resource to note that CD is distinct from a food allergy:

It isn’t a food allergy or an intolerance. It is an autoimmune disease. This means the good healthy cells in my body, get destroyed, by my own bodyguard cells. My bodyguard cells can’t stop the bad proteins. These are found in food like wheat, rye, barley, oats, and malt, which is called gluten. The “gluten” sneaks into the lining of my small intestine […] eats all the good food and nutrients [55].

Some books however, included very little explanation regarding CD or gluten, such as *How Hayden Found Her New Favourite Foods*, which only states: “Until the doctor told her to eat only gluten-free foods” [60] with no information on what gluten is or what foods this might include. *Silly-Yak* skipped this stage entirely. Catarino et al. found that achieving knowledge about a disease is the foundation to acquiring the necessary tools to manage a diagnosis [25]. Without this information, the understanding of why CD treatment is necessary is not developed.

Several of the resources in this study show a lack of specificity in the information provided, including how a child is diagnosed and tested for CD. For example, *Gluten Tootin’* describes the testing process by stating that the character, Ben, “needed to find some answers, to know who or what to blame, so off to the doctor he went, with his mum to find out why […] she poked and prodded young Ben, then grinned and said … ’I know why’” [61]. If children are to prepare for CD testing, it is unhelpful to suggest that the process requires only a bit of “poking and prodding”. This could make some children feel that the more invasive process they may have been required to go through for their own CD testing was not right or fair. Thus, it would likely be more beneficial to include the diagnosis process to normalise this for every child in an age-appropriate way. Existing literature has shown that using an outlet such as picture books is effective in helping to normalise living with a chronic disease for children [20] and increase health information so children can efficiently manage their illness [17]. However, that information needs to be accurate, or normalisation is at risk of becoming isolation. Some resources included more information in this respect than others; however, this was still minimal. *Sophie Doesn’t Eat Gluten* included that “the doctor took blood and tested it” [54], and *Celia’s Coeliac Story* mentioned that “Celia will need a blood test” [56]. *I Have Coeliac Disease: Autumn’s Story* was the only resource that explained what a blood test reveals: “I had to have a blood test. This showed I had a lot of antibodies (proteins) in the tissue of my small intestine” [55].

Some of the resources included some incorrect information which could lead to children not accurately following the GFD. In *Little Jak the Silly Yak: The Apple Pie Quest*, Jak meets a cow named Brie who is lactose intolerant. Brie suggests a good cafe to try since she “loves their oaty lattes. And their freshly baked oat cookies!” [58], a statement which could be confusing for children with CD as oats typically contain gluten. There is also a lack of specificity provided in the range of symptoms someone with CD could experience: *How Hayden Found Her New Favourite Foods* merely states that “her tummy hurt” [60] and that is why Hayden went to the doctor, while *Sophie Doesn’t Eat Gluten* states that “gluten makes her feel unwell” [54]. This vague description could result in a poor understanding of why it is necessary to miss out on tasty foods that everyone else can eat and how CD is unique from the many other conditions that can result in stomach upset. This could also make it difficult for children to understand the true symptoms of their illness and thus develop accurate bodily attunement. As research has shown, there is a wide range of symptoms that eating gluten can produce for those living with CD [9,10]. *I Have Coeliac Disease: Autumn’s Story* is the only resource to provide a more extensive range of possible symptoms someone with CD could experience: “Some days I feel very sick […] I get diarrhoea. Coeliac children can also suffer from weight loss. Sometimes it is a bloated and painful tummy or I don’t feel like eating anything” [55]. Furthermore, none of the resources include any information regarding the long-term impacts associated with CD. Considering that over 11% of children with CD also have no noticeable symptoms [13], this would also be beneficial to include for further awareness of the range of possibilities associated with this illness. With the strong connection between untreated CD and long-term mental and physical health concerns [12,15], being well-informed is crucial for overall health and wellbeing and further highlights the importance of early diagnosis and complete GFD adherence [11].

### 4.2. Promotion of CD Management

This theme, while providing similar intentions to Gildersleeve et al.’s theme of promotion of a new normal [46], provided a stronger message for CD specifically. This theme addresses how a young child with CD can comply with a lifelong treatment of adhering to a GFD. Being prepared and flexible daily and being able to handle the challenges with which they are faced is vital for consistent adherence to a GFD and for providing a basis in reducing hypervigilance and anxiety. Knowing where and how to find safe food is typically the greatest challenge in adhering to treatment for CD [27,31]. As such, some of the books include types of foods that can be consumed or advice on how to make favourite foods gluten free. *I Have Coeliac Disease: Autumn’s Story* explains: “I can eat fresh fruits, vegetables, milk and eggs. Also unprocessed meats and poultry, rice, corn and potatoes” [55], and *Little Jak the Silly Yak Has Silly-Yak Disease* narrates:

But do not worry, little Jak, there’s plenty you CAN eat. Like carrots and broccoli, corn on the cob, potatoes, rice, and meat. And if you fancy pizza or pie, there’s plenty you can make. Just use gluten-free biscuits in a gluten-free cheesecake. Switch out normal plain flour for grains like corn or rice. You’ll find that you can make it all, and it actually tastes nice [57].

Several of the resources ignored the promotion of gluten-free food accessibility, instead focusing on the taste of gluten-free food as the primary obstacle to a GFD. These resources fail to acknowledge what to do, for example, when away from home and the difficulty in accessing gluten-free food. In *Gluten Tootin’*, the only information provided to advise children how they can manage their diagnosis is: “So now Ben knew what he must and mustn’t eat it wouldn’t be easy, but he promised not to cheat. He said no to wheat in bread, cake and noodles and found lots of tasty food he liked, even vegataboodles” [61]. *Celia’s Coeliac Story* also includes very limited management promotion, noting only that “there was so much choice that Celia was in awe” [56], along with a selection of gluten-free recipes in the end of the book. The book *Silly-Yak* suggests a GFD is terrible, without any alternative: “What he likes to munch, crunch and chew, is not made from something new. Jack’s food isn’t the best, it crumbles and falls and makes a mess. It is all so hard and dry and new. Jack doesn’t like this different food” [62]. To consistently state the food children with CD must eat for the rest of their lives does not taste good is not reassuring and unlikely to help the child cope with their diagnosis and its treatment. It could also prime the idea that gluten-free food tastes bad, which for some children will stop them from eating it before even trying it for themselves. Promoting positive aspects of behaviour change instead of focusing on the negatives is how young children learn positive/negative behaviours [65].

Some of the books include gluten-free recipes, providing ideas for children and parents. Indeed, the plot of *How Hayden Found Her New Favourite Foods* focuses on Hayden finding recipes that she can make gluten free. This book shows Hayden and her parents finding lots of new recipes to try and having fun doing so:

We found a way to make them gluten-free, remember? They taste really good […] They were so delicious that Hayden and her mum ate the whole batch at once […] Hayden asked her dad if they could try Ashley’s recipe for American barbecue ribs […] and basted the ribs in gluten-free sauce while Hayden made the salad […] That was one of the tastiest things I’ve ever eaten, even when I wasn’t gluten-free [60].

The three resources that provide appropriate instruction on gluten cross-contamination educate children with important and specific knowledge. This is vital instruction to follow due to the harm that even a small amount of gluten can have on someone with CD [6]. *I Have Coeliac Disease: Autumn’s Story* reminds the reader that “even just a breadcrumb with gluten will make me sick. Cooking utensils need to be kept separate during food preparation and cooking. Wash surfaces thoroughly and use separate breadboards” [55], while *Little Jak the Silly Yak Has Silly-Yak Disease* states: “and don’t forget when cooking, if gluten is nearby, make sure the food for you young Jak, stays well away, goodbye” [57].

Several of the resources suggest getting help from adults, mostly from the mother, instead of promoting necessary and age-appropriate skills for the child to develop for themselves. While this advice of asking can sometimes be necessary, children also need to build independent skills to keep themselves safe. For example, in *Sophie Doesn’t Eat Gluten*:

If a packet says gluten free, Sophie knows she can eat that food without getting ill. When Sophie is older, she will check foods herself […] Adults keep Sophie safe […] reading the packets. Sophie asks an adult if she isn’t sure […] if someone gives her food, she asks an adult to check that it’s gluten free [54].

*I Have Coeliac Disease: Autumn’s Story* also narrates: “I have to get mum, dad, or an adult to read all the labels on food and drinks before I have them” [55]. While it is appropriate and important for children to know that adults are there for support if needed, this does suggest this will remain the parent’s responsibility instead of providing the tools for children with CD to eventually manage their disease themselves, which may lower children’s self-efficacy and foster dependency and vulnerability as well as lower bodily attunement. As Meyer and Rosenblum found, while adhering to and management of a GFD is partly a parental responsibility for children of this age [21], helping children acquire these skills to self-manage is vital. Younger children are also more likely to comply with adherence than older children [29,30]; therefore, this is a fitting age to begin to learn how to be responsible for a lifelong health condition.

### 4.3. Anxiety and Hypervigilance Reduction

Aligning with the Gildersleeve et al. model [46], the CD picture books followed a similar pattern of moving through a cycle from promoting management of a diagnosis into the difficulties a diagnosis can bring. The potential negative consequences of being unprepared or unable to adapt to the possibilities of being in a situation where gluten-free food is unavailable or difficult to find can lead to heightened levels of anxiety and hypervigilance [35,37]. Acknowledging these difficulties and providing children and families with the practical tools necessary for managing a CD diagnosis can help reduce these fears. As Schoppmann et al. found, picture books are an effective resource for helping validate children’s feelings and understanding their emotions and concerns [4]. This theme is therefore informed by the two subthemes of Validating Feelings and Reducing Concerns.

#### 4.3.1. Validating Feelings

The literature found that even before being diagnosed with CD, related psychological issues such as depression and anxiety can be of major concern for children [9], and following a diagnosis, lifelong adherence to a GFD is challenging in many ways [29,35]. Therefore, validating how these challenges can make a young child feel is vital for QOL and coping. Only a few of the resources explicitly recognise that feeling unwell is frustrating or that following a GFD is challenging. *Sophie Doesn’t Eat Gluten* states only that “sometimes Sophie feels sad that she can’t eat the same thing as her friends” [54], while *I Have Coeliac Disease: Autumn’s Story* mentions that “it is hard to say ‘no, thank you’ some days” [55]. *Little Jak the Silly Yak Has Silly-Yak Disease* includes:

I’m feeling pretty scared [ …] But what am I going to eat? I really do love bread. Little Jak let out a cry: this has been the worst day! I can’t have any food that I like, or my tummy will just roar! But mummy please, this isn’t fair. I don’t want this anymore [57].

These resources attempt to include a level of understanding for the challenges young children with CD are going through; however, the validation suggested within these resources is focused on a shallow level of understanding by suggesting that the main difficulties are, again, that gluten-free food does not taste good, already identified as an unhelpful discourse.

However, *Little Jak the Silly Yak at Tara’s Birthday Bash* does observe from Jak’s perspective: “Would there be food for him? He started to shrink […] If there’s no gluten-free food for me, I’ll be sad. Jak sat down in a slump with a sigh. But what if the tasty treats all contain rye?” [59]. This aligns with the literature that observes that going to a social event with the possibility of nothing safe to eat is an anxious task [9,28]. What is missing from all of these resources is the validation that coping with a lifelong diagnosis is incredibly difficult. There is never a break from the constant vigilance and ongoing checking of foods, having to be careful of possible gluten exposure, and always being prepared for food insecurity [28,29,35]. Validating the fact that this diagnosis is never cured and how difficult this is to cope with [28] is a powerful message required for a successful CD picture book and is not achieved within any of the resources analysed in this study.

#### 4.3.2. Reducing Concerns

After the child’s feelings have been validated, providing coping strategies and positive ways to reduce concerns is necessary for children and families learning to adapt and manage traumatic health concerns [46]. Unfortunately, reassurance of a happy ending within several of these resources suggests that because gluten-free food can still taste nice, any concern has been addressed. *Sophie Doesn’t Eat Gluten* attempts to reduce anxiety since there are “lots of foods that Sophie can eat […] Sophie feels happy that she gets to eat yummy gluten free cakes and bread [… and she] gets to go to a special gluten free aisle” [54], while *How Hayden Found Her New Favourite Foods* observes: “I thought it was going to be horrible when I found out I had coeliac disease. I thought I wouldn’t like the food I was allowed to eat. But then I found out being gluten-free can be awesome” [60].

Practical coping strategies are provided within two of the resources, which can assist children to help themselves feel better. As Sady et al. found, educating children how to resist regular (non-gluten-free) food as a source of temptation and not giving into the impulsivity young children often exhibit is one of the greatest challenges for children of this age [31]. Therefore, providing strategies to plan for difficult situations where gluten-free food may be inaccessible is fundamental. *Little Jak the Silly Yak at Tara’s Birthday Bash* suggests bringing gluten-free food along to a birthday party so Jak will not miss out on being able to eat party food with his friends: “They spent time together, preparing the treats. Sandwiches, cupcakes and even some sweets. Oh, thank you, mummy. These treats look so great. I can’t wait until I can fill up my plate” [57]. In *How Hayden Found Her New Favourite Foods*, Hayden and her mother reach out to an online CD community to find gluten-free recipes.

At the end of *Gluten Tootin’*, the protagonist, Ben, is depicted as a superhero as he is so brave for eating a GFD. The superhero trope suggests that children must be extraordinarily brave to cope with a chronic illness, thereby creating an unrealistic ideal to which children must aspire [66]. The book thereby suggests that children are strong and brave for coping; however, everyone has moments when they do not feel like being strong and brave. This is part of the challenge with living with a chronic condition—that there is never a chance to have a rest from the constant management and challenges [28]. Insisting on behaving as a superhero thus erases the acknowledgment of that response.

### 4.4. Community and Connection

This theme builds on the literature that suggests that young children learning to manage a chronic illness require the help of friends, family, and other adults to assist them in keeping safe by reinforcing health messaging [30], which helps build a sense of connection and belonging. *Sophie Doesn’t Eat Gluten* observes that “Sophie’s friends help to keep her safe as well” [54]. In *How Hayden Found Her New Favourite Foods*, Hayden is able to gain help and support from her newfound friends from the online CD community:

Everyone was friendly and helpful […] Hayden realised she’d been chatting to her new friends with her mum all morning. She’d learnt so much about them and the yummy gluten-free food they ate […] Hayden was really enjoying sharing photos of her cooking with her friends in the cooking club. Everyone had been helping with recipes and telling her how amazing her cooking looked [60].

These resources provide a sense of connection through seeking advice and support from peers, suggesting that managing a CD diagnosis is easier with the help of others.

However, several of the resources fail to build upon the importance of the community as a support system, instead solely suggesting that parents and other adults are there to manage the responsibility of adherence for the child, which also underlines the need for individual agency.

The importance of Community and Connection is strengthened by literature that suggests GFD adherence is lower when children are at school [67]. Therefore, the greater the support and understanding provided by teachers and peers, the greater chance of children feeling equipped to manage their illness [25]. In *Little Jak the Silly Yak at Tara’s Birthday Bash*, Tara and Jak’s other friends at the party show inclusion of Jak within their friendship group by making all of the party food gluten-free. While this promotes a strong sense of Community and Connection, providing all gluten-free food at the birthday party for everyone may set up an unrealistic expectation for children with CD. It is not always possible, and potentially quite unlikely, that all gluten-free food at a child’s birthday party would be provided due to various reasons (e.g., cost, time). Nevertheless, since feeling different from peers and experiencing consequent social isolation can be a consequence of CD [35], having friends and family to help make children with CD feel accepted is crucial to the child’s coping. The importance of inclusion is further portrayed in *How Hayden Found Her New Favourite Foods*, which provides a sense of empathy and understanding for the challenges CD can bring. Hayden’s grandmother brings a gluten-free chicken pie made from scratch, which she describes as being “made with love”. The grandmother continues by saying “you look so well, and happy too […] are you getting used to having coeliac disease?” [60]. Grandparents are a significant influential figure in a child’s life; however, they can also tend to be a source of dismissal when it comes to the acknowledgement of diet and allergies due to generational misunderstandings; therefore, portraying acceptance and support from a grandparent is an important site of connection for CD management.

All of the resources, designed with colourful and fun pictures, were likely created with good intentions to produce helpful, developmentally appropriate resources for children with CD; however, several of the resources fall short of providing necessary information children and their parents require to effectively self-manage. The three books in the *Little Jak the Silly Yak* series together do a better job at fulfilling most of the criteria compared to the other resources; however, on their own, each book is lacking vital information. The three books would need to be purchased as a set to work as a sufficiently inclusive resource.

This study identified three main limitations. The first potential limitation is reliability. As qualitative analysis is based on interpretations, these can be heavily influenced by the researcher and their experiences. Due to the purposeful reflexive nature of thematic analysis, personal experience and knowledge can influence observations and findings. The researcher’s own subjectivity may have limited the consistency and reliability of the findings. This subjectivity may have led to variations in the analysis, where different researchers might have identified different interpretations or themes within the same dataset; however, this may also offer unique and nuanced insights. Through following Braun and Clarke’s recommendations for RTA, potential bias was minimised and potential for further reliability was increased [52].

The second limitation is the modest dataset used for analysing and interpreting the findings, albeit appropriate for the RQs and selected analyses. This was mostly due to the available resources that fit the inclusion criteria. Due to the lack of understanding of the severity of CD, most of the resources were also written by individuals with their own lived experience of CD or with children diagnosed with CD; therefore, most of the resources in this study were self-published. Expanding the inclusion criteria to including picture books from outside Australia and the UK, there may be a greater number of available resources that have been published at reputable publishing houses. This could potentially offer new insights regarding the findings from this study.

Finally, not only was the dataset on the modest side, the dataset also consisted solely of books written in English. This highlights a lack of cultural diversity within the included resources and suggests a caution for generalising findings from this study to other ethnicities on an international scale. As mentioned above, the availability of resources within the inclusion criteria was discrete and further emphasises the need for exploring a wider range of texts and texts that are made available in multiple languages.

## 5. Conclusions

The findings of this study have highlighted the lack of and the necessity for adequate and more appropriate CD picture books and narrative resources. Existing research has highlighted the impact a diagnosis of CD has on young children and their families [6,28,35], and the current study has reinforced the scarcity of available resources providing essential and accurate CD knowledge and coping strategies. This research is novel in examining existing CD resources and has provided an ideal opportunity to further explore the MCN specific to CD developed in this study and the potential for future research. It is hoped that these findings might benefit health professionals or health organisations to develop a more ideal and beneficial narrative resource appropriate for young children diagnosed with CD through utilising the CD-specific MCN. It might also be valuable to extend this research into developing different mediums and modalities that are appealing to children, such as television promotions, informational posters or leaflets, and other multimedia outlets, as well as to consider strategies to support children who require additional communication support. This could help to further educate and spread the message in a range of avenues for further inclusivity as well as form the basis of discussions between children and caregivers, educators, or peers [68]. Further understanding of the settings within which such reading takes place and how this might impact coping would support the present study. It is hoped that this study might offer further insight and understanding for health professionals, clinicians, and educators as well as be utilised in clinician training as a therapeutic and health promotion intervention, in support within schools for educators and students, by other authors of similar books for children, in coping methods for individuals and families, and in exploring the relation to other health conditions to promote health literacy and support around CD.

This study aimed to address the gap in the literature focusing on effective coping strategies for young children and their families dealing with a CD diagnosis. The overwhelming evidence base that this project has emphasised regarding the lack of understanding and support available for young children diagnosed with CD highlights the crucial need for a well-developed resource. Through analysing the resources in this study, it became evident that the included CD picture books stop short of providing accurate and specific information and coping strategies and fail to focus on the difficulties that require attention. As children’s stories are a highly utilised resource for helping parents explain to their children how to cope with specific topics and issues, the lack of appropriate resources may leave families with nowhere to turn. Although resources are available for children and their families coping with a CD diagnosis, they remain lacking in content appropriate for delivering a well-developed health narrative. Further development of CD resources is required to maximise the potential benefit of children’s books as a health promotion tool, and this research has provided an outline of how these resources could potentially be further explored.

## Figures and Tables

**Table 1:** List of included CD picture books.

Title	Author	Lived Experience	Publisher, Country, and Year	Endorsements	Ideal Reader
*Sophie Doesn’t Eat Gluten: A Book About Coeliac Disease* [54]	Cressida Bullock	Author diagnosed coeliac	Independently Published England, UK 2022	No mention	Children; families; classrooms
*I Have Coeliac Disease: Autumn’s Story* [55]	Vivienne Marie	Author’s child diagnosed coeliac	CreateSpace Independent Publishing Platform England, UK 2017	No mention	Children; teachers; family; peers
*Celia’s Coeliac Story* [56]	Christiana Botziou	Author diagnosed coeliac	Independently Published UK 2019	No mention	Children; parents
*Little Jak the Silly Yak Has Silly-Yak Disease* [57]	Jasmine Quarless-Carew	Author diagnosed coeliac	Independently Published UK 2023	No mention	Children
*Little Jak the Silly Yak: The Apple Pie Quest* [58]	Jasmine Quarless-Carew	Author diagnosed coeliac	Independently Published UK 2023	No mention	Children
*Little Jak the Silly Yak at Tara’s Birthday Bash* [59]	Jasmine Quarless-Carew	Author diagnosed coeliac	Independently Published UK 2023	No mention	Children
*How Hayden Found Her New Favourite Foods* [60]	Stefi Mac	Author’s child diagnosed coeliac	Wolfebaun Press Australia 2023	Endorsed by Coeliac Australia and Allied Health Professionals	Children; families
*Gluten Tootin’* [61]	Kylie Anderson	Author’s child diagnosed coeliac	PEG Books Australia 2020	Endorsed by Coeliac Australia	Children; parents; specialists
*Silly-Yak* [62]	Alexandra Rose Mangano	Author diagnosed coeliac	InHouse Publishing Australia 2021	No mention	Children; families; schools

**Table 2:** Characteristics of CD picture books against Gildersleeve et al.’s MCN [46].

	Characteristics			
Picture Book Title	Information Provision	Promotion of a New Normal	Anxiety Reduction	Community and Connection
*Sophie Doesn’t Eat Gluten: A Book About Coeliac Disease* [54]	Sore tummy as a symptom; diagnosis; what is gluten	What foods are GF; label reading; basic adherence	Feels sad sometimes but yummy GF foods make her happy	Adults help to check food; friends help keep her safe by not sharing food
*I Have Coeliac Disease: Autumn’s Story* [55]	What is CD; symptoms; genetics; diagnosis; treatment	Adherence; cross-contamination; label reading; being prepared	Hard some days to say “no”; gets easier with time	Adults help to check labels; telling schools and teachers
*Celia’s Coeliac Story* [56]	Symptoms; diagnosis; what is CD; treatment; GF recipe	How to make GF food exciting	Hope for feeling better; lots of GF choice	
*Little Jak the Silly Yak Has Silly-Yak Disease* [57]	What is CD; symptoms; treatment; GF recipe	Adherence; label reading		Reader identity
*Little Jak the Silly Yak: The Apple Pie Quest* [58]	What is CD; basic symptoms; GF recipe	Minimal adherence; cross-contamination	Difficulty finding GF food; helps increase hope that food can still taste good and is available	It is important to connect with similar peers
*Little Jak the Silly Yak at Tara’s Birthday Bash* [59]	Minimal what is CD; tummy could hurt; GF recipe		Feeling sad about the possibility of no food	Inclusion with friends and family
*How Hayden Found Her New Favourite Foods* [60]	Tummy hurts from eating gluten	Adapting to a new diet; plenty of GF recipes available	Unfair to not eat what everyone else can; find alternate foods or make favourite foods GF; not sick anymore when eating GF	Connecting with online GF kids club; help from new friends who understand; help from mother
*Gluten Tootin’* [61]	Sore tummy; mention of embarrassing symptoms; mention of going to doctor for diagnosis only; what cannot be eaten	Lots of tasty GF food	Acknowledges it is not easy; however, tummy is happy and healthy without gluten	
*Silly-Yak* [62]			Suggests that with a lot of effort, good food can be found	

## Data Availability

The original contributions presented in the study are included in the article. Further inquiries can be directed to the corresponding author.

## Abbreviations

The following abbreviations are used in this manuscript:

CD	Coeliac disease
GFD	Gluten-free diet
QOL	Quality of life
MCN	Model of coping narratives
